# The Social Contagion of Generosity

**DOI:** 10.1371/journal.pone.0087275

**Published:** 2014-02-13

**Authors:** Milena Tsvetkova, Michael W. Macy

**Affiliations:** 1 Department of Sociology, Cornell University, Ithaca, New York, United States of America; 2 Department of Information Science, Cornell University, Ithaca, New York, United States of America; Hungarian Academy of Sciences, Hungary

## Abstract

Why do people help strangers when there is a low probability that help will be directly reciprocated or socially rewarded? A possible explanation is that these acts are contagious: those who receive or observe help from a stranger become more likely to help others. We test two mechanisms for the social contagion of generosity among strangers: generalized reciprocity (a recipient of generosity is more likely to pay it forward) and third-party influence (an observer of generous behavior is more likely to emulate it). We use an online experiment with randomized trials to test the two hypothesized mechanisms and their interaction by manipulating the extent to which participants receive and observe help. Results show that receiving help can increase the willingness to be generous towards others, but observing help can have the opposite effect, especially among those who have not received help. These results suggest that observing widespread generosity may attenuate the belief that one’s own efforts are needed.

## Introduction

“*In the order of nature we cannot render benefits to those from whom we receive them, or only seldom. But the benefit we receive must be rendered again, line for line, deed for deed, cent for cent, to somebody*” – Ralph Waldo Emerson.

*“…[W]hen you meet with another honest Man in similar Distress, you must pay me by lending this Sum to him; enjoining him to discharge the Debt by a like operation, when he shall be able, and shall meet with another opportunity. I hope it may thus go thro’ many hands, before it meets with a Knave that will stop its Progress.*” – Benjamin Franklin, to a stranger whom he had given money.

On a cold December morning in 2012, in the drive-through of the Tim Hortons in Winnipeg, Canada, a stranger generously picked up the tab for the coffee order of the next customer waiting in line. That person paid the bill of the next stranger in line. And so did the following 226 customers [1]. The practice of “paying it forward” spread not only to other customers of the restaurant but to other restaurants – the Chick-fil-A drive-through off Highway 46 in New Braunfels, Texas, a Dunkin’ Donuts drive-through in Detroit, and a McDonald’s drive-through in Fargo, North Dakota [2],[3]. “Serial pay-it-forward incidents involving between 4 and 24 cars have been reported at Wendy’s, McDonald’s, Starbucks, Del Taco, Taco Bell, KFC and Dunkin’ Donuts locations in Maryland, Florida, California, Texas, Louisiana, Pennsylvania, Oklahoma, Georgia, Alabama, North Dakota, Michigan, North Carolina and Washington.”

“Pay it forward” is not limited to restaurant drive-ins. Acts of generosity occur commonly in daily life, ranging from anonymous blood donations to stopping to help a stranded motorist. In online communities, voluntary contributions are pervasive: every day, millions of people write restaurant reviews, leave product ratings, provide answers to an unknown user’s question, or contribute lines of code to open-source software, all without any direct reward or recognition. Why, in the absence of external sanctions and opportunities for reciprocation, do people help strangers?

One possible explanation is that helping is driven by receiving or observing help. In other words, generosity towards strangers may be socially contagious. In a ground-breaking study, Fowler and Christakis [4] found evidence that generous behavior can indeed ripple through social networks. In particular, the authors showed that the “three degrees of influence” rule observed for other contagions, such as the spread of happiness and obesity [5], applies as well to generous behavior. If you help someone, you not only increase the likelihood that they help others, but that those they help will also help others, and so on, out to three steps. Using similar experimental designs, Suri and Watts [6] and Jordan et al. [7] also found that generous behavior was contagious, but that it does not spread beyond the direct interaction.

The contagiousness of generosity may depend on the mechanism by which it spreads. Fowler and Christakis [4] and Suri and Watts [6] tested the spread of generosity on networks but their studies were not designed to identify the underlying mechanisms. They used a public goods experiment in which multiple individuals donate to a common pool and then share the investment equally. Contagion occurs when an individual who has interacted with generous partners in one group donates more in the next group. Although useful in demonstrating contagion, the public-goods experimental design, including the *N*-person Prisoner’s Dilemma [7], [8], does not distinguish between receiving and observing generosity since group members also benefit from the generous acts they observe. The present research uses an innovative experimental design to distinguish between the two processes and to measure their contribution to the contagion of generosity.

### Generalized Reciprocity and Third-Party Influence

**Figure 1:**
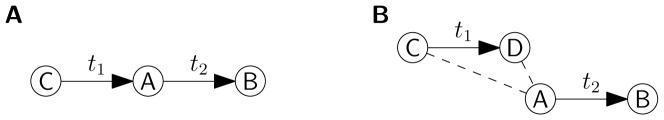
Two mechanisms for the contagion of generosity. (A) Generalized reciprocity: *A* helps *B* because *C* has helped *A*. (B) Third-party influence: *A* helps *B* because *A* has observed *C* help *D*. Arrows indicate helping or giving, dashed lines indicate observing.

Previous research suggests that there are two distinct mechanisms for the social contagion of generosity among strangers: generalized reciprocity (GR) and third-party influence (TPI). Generalized reciprocity (GR) refers to cases in which those who benefit from the kindness of strangers become more generous towards others in the future. As diagramed in Figure 1, A helps B because C has helped A [9], [10]. Third-party influence (TPI) refers to cases in which those who observe kindness between strangers become more generous towards a stranger: A helps B because A has observed C help D. GR characterizes “pay it forward” behavior triggered by normative or expressive responses to being helped [11], while TPI characterizes social learning through imitation of others’ behavior.

The difference in the two mechanisms parallels Deutsch and Gerard’s [12] distinction between normative and informational influence. GR is driven by an “injunctive norm” [13], [14] – a normative obligation to express one’s gratitude at being helped not by repaying the helper but by acting as the helper acted. TPI is driven by a “descriptive norm” – to follow the example of others’ behavior when unsure about how one is expected to act.

GR and TPI also differ in the pattern of transmission. GR transmits the contagion from person to person through direct contact and hence its contagious effect is limited to the one person who was previously helped. In contrast, TPI has the potential to broadcast the contagion from one person to any number of observers. For example, when a stranger stops to help a stranded motorist, only one person receives help but thousands of passersby might observe helping behavior.

**Figure 2:**
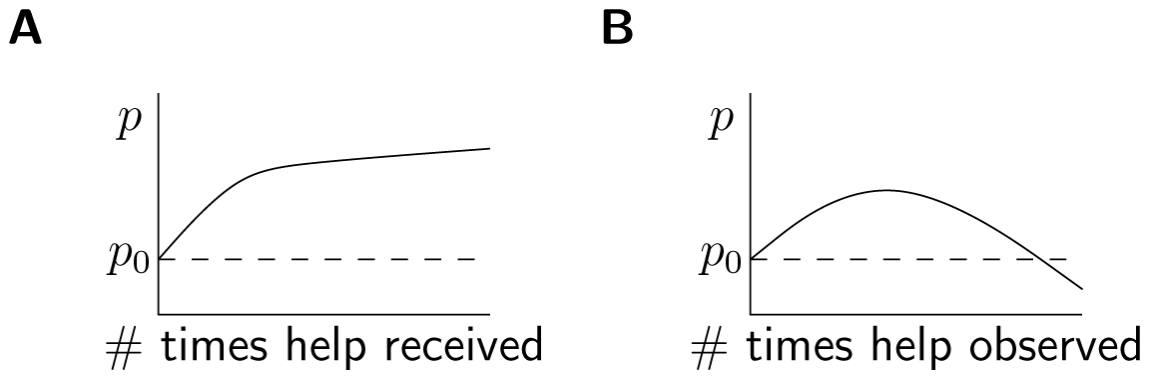
Monotonic and non-monotonic changes in the probability to help. Both (A) generalized reciprocity and (B) third-party influence are expected to increase the probability to help (*p*) above the baseline level of “unconditional generosity” (*p*_0_) but the effects from repeatedly receiving and observing help are expected to differ.

This multiplier effect of TPI means that we are far more likely to observe generosity than to receive it. If widespread observation establishes a descriptive norm that in turn makes each individual more likely to be generous, then TPI could generate a powerful self-reinforcing dynamic [15]. However, previous research on threshold models of social contagion [16]–[19], the “free-rider” problem in collective action [20], social loafing in groups [21], the Volunteer’s Dilemma [22], the “bystander effect,” and the diffusion of responsibility [23] all point to a very different possibility: that an individual is more likely to help or contribute when confronted with the stark reality that “if you don’t do it, nobody else will” [20]. Once a descriptive norm has been established and people take for granted that someone else is likely to help, one’s own contribution appears less essential. In short, once the observed level of generosity is sufficient to safely assume that one’s own contribution is not needed, the positive effect of the descriptive norm can be expected to reverse, such that third-party influence becomes negative (i.e. the observer does the opposite of the observed behavior; see Figure 2).

Although conceptually distinct, GR and TPI are not proposed as alternative explanations for the contagion of generosity among strangers. Rather, the two mechanisms are likely to interact, due to the greater likelihood to both receive and observe generosity from strangers in populations where this behavior is normative. When people observe helping behavior after previously receiving help from a stranger, the normative influence from GR is expected to mitigate the negative effects of observing widespread acts of helping.

The present study aims to test GR and TPI as possible mechanisms in the social contagion of generosity. This requires an experimental design in which receiving and observing generosity are not confounded by each other or by the effects of closely related mechanisms. In particular, GR can be confounded by indirect reciprocity and TPI by peer pressure, and both GR and TPI may be confounded by unconditional generosity. In the sections that follow, we elaborate the distinctions, both theoretically and operationally.

### Generalized Reciprocity vs. Indirect Reciprocity

Generalized reciprocity should not be confused with indirect reciprocity. Both involve the pattern depicted in Figure 1 in which A helps B and C helps A, but they differ in sequencing, and the difference in temporal ordering implies different motivations. With GR, C helps A before A helps B, while with indirect reciprocity, C helps A after A helps B. GR is more plausibly motivated by feelings of obligation and/or gratitude in response to receiving help, while indirect reciprocity is generally assumed to be instrumentally motivated as a reputational strategy for obtaining help [11], [24], [25].

Generalized reciprocity also differs from generalized exchange [26]. The latter refers to a pattern of exchange between two members of a group, both of whom give and receive from a group member but not necessarily one another. By that definition, both GR and indirect reciprocity can be classified as two different forms of generalized exchange.

Prosocial behavior could increase when reciprocity is generalized as well as when it is indirect, but only the former leads to social contagion through transmission upon contact. With GR, the helping behavior is backward-looking – a response to the helping behavior of others. In contrast, when reciprocity is indirect, the helping behavior is forward-looking, in anticipation of the receipt of help. Indirect reciprocity could increase generous behavior because it changes the interaction situation by modifying the incentives. GR could increase generous behavior because generosity generates more generosity. Unfortunately, observational studies of generalized exchange cannot distinguish between GR and indirect reciprocity. For example, the three best documented cases of generalized exchange in naturally occurring environments – the Kula trading ring among South Pacific islanders [27], the kinship relations among aboriginal tribes [28], and the support networks of low-income black women [29] – involve very small communities, in which helping behavior could be motivated by anticipated rewards rather than as a response to being helped. Similarly, generalized-exchange experiments cannot distinguish GR and indirect reciprocity if interactions are repeated in fixed network structures and/or with full information about others’ behavior [30]–[33].

The effects of GR can be isolated from possible confounding effects of indirect reciprocity by keeping interactions anonymous and by preventing anyone else from knowing about an actor’s past behavior. For example, [34] and [10] isolate GR from indirect reciprocity by using anonymous one-shot interactions that remove opportunities for reputation-based rewards.

### Third-party Influence vs. Peer Pressure

Like GR, TPI can also be confused with other types of third-party effects. The TPI we refer to corresponds to what Deutsch and Gerard [12] call “informational influence,” in which an actor models an observed behavior. Deutsch and Gerard distinguish this from “normative influence,” in which an actor engages in a behavior that is socially approved. When influence is normative, one conforms to others’ behavior in order to be liked and accepted. When influence is informational, the actor conforms to a descriptive rather than prescriptive norm. For example, in “rational herding” [35], conformity occurs because one assumes that others know better what the appropriate behavior should be. The two types of influence are associated with different types of social relationships. Normative social influence (or “peer pressure”) depends on the desire for social approval from significant others, which in turn is likely to be greater when there is a pre-existing and on-going social relationship, such as that between family members, friends, or colleagues. In contrast, when relationships are novel and/or transient, as when interacting with strangers, dependence on cues from network neighbors may be more important than dependence on social approval.

This distinction between normative and informational influence is therefore important for the study of generosity among strangers. Normative influence is more relevant for the enforcement of pro-social behavior in tight-knit social groups whose members depend on one another for social approval, while informational influence is more relevant for the contagion of generosity among strangers. Most previous studies of social contagion have been observations of cascades passing through pre-existing social relationships between people who already knew one another [5], [36]. These situations are not well-suited for the study of informational influence, which is likely to be obscured and confounded by normative pressures. The effects of informational influence can be isolated from possible confounding effects of peer pressure by keeping all actors anonymous and precluding repeated local interactions. For example, Salganik, Dodds, and Watts [37] succeed in detecting informational influence in a cultural market by letting participants interact a single time and by revealing to them only the aggregated behavior of others.

### Unconditional Generosity as Baseline

In addition to distinguishing GR from indirect reciprocity and TPI from normative influence, it is crucial to also distinguish both GR and TPI from another important and possibly confounding mechanism – unconditional generosity. Unlike GR and TPI, unconditional generosity occurs when A helps B even though A has not received help from C nor observed C helping D. Thus, when A helps B after receiving help from C, it is possible that A would have helped B anyway. This possibility was overlooked by two previous studies of GR [10], [34]. These studies offer evidence that individuals who have been recent recipients of generosity are likely to be similarly generous to a third party, even if they know that they cannot benefit from this in the future. However, it is unclear whether participants would have made a similar donation even if they had not received a donation from a stranger. In other words, the observed generosity could have been due to unconditional generosity, rather than the result of contagion through GR.

The effects of GR and TPI can be isolated from possible confounding effects of unconditional generosity by measuring the effect of receiving and observing generosity above and beyond a baseline tendency to help under an otherwise identical decision situation but in which help is neither received nor observed. Similarly, the effects of GR can be isolated from TPI by measuring the effect of receiving help among those who are unable to observe helping behavior more generally. These conditions rarely obtain in natural settings, which limits the ability to identify the underlying mechanisms in observational studies of helping behavior. We therefore designed and conducted an experiment with human participants.

## Materials and Methods

### Ethics Statement

The research was approved by the Institutional Review Board for Human Participants of Cornell University. Written informed consent was obtained from all subjects.

### Procedure

Subjects were recruited from and paid through the online crowdsourcing platform Amazon Mechanical Turk [38]–[42] but interacted on a website hosted on our webserver. The study was designed as a sequential two-player investment/gift-exchange game in groups of 150 with random partner selection. In the game, a participant could choose to return part of their payment so that another anonymous participant could benefit (similarly to [10] and [31]).

The study was conducted in March–April, 2013. We first recruited a pool of potential participants by posting a task on the online crowdsourcing platform Amazon Mechanical Turk (AMT). The task was called “Sign up to participate in the Invitation Game” and paid $0.20 when submitted. The task invited AMT users to sign up for a study that offered the chance to earn up to $14–21 for doing the same $2–3 ten-minute task multiple times. To sign up, an AMT user simply needed to read and agree to the terms of the study and provide standard demographic information (gender, age, ethnicity, nationality, education, religious affiliation, and income). The instructions emphasized that the demographic information would not be used for selecting the participants. The AMT users were informed that they could only participate in the task and earn the promised amount if they were randomly selected from the pool of potential participants. Participants were eligible to be selected multiple times but there was no guarantee that they would be selected even once. If selected, the participant was to receive an e-mail notification with further instructions. (See the recruitment instructions in Experiment Instructions S1.).

The email invitation informed recipients that they were randomly chosen to participate in the Invitation Game, which they had to complete within 24 hours. Participants were given their AMT worker ID and a unique randomly generated Invitation ID to log into our website. On the website, participants read a description of the Invitation Game, answered five multiple-choice questions testing their understanding of the game rules, wrote a short summary of the decision situation they were facing, and made a single decision about whether to donate money to benefit a stranger (see Experiment Instructions S1).

The game description explained to each participant that they would be paid the amount promised in the original solicitation, which included a “base” payment plus a “bonus” payment. Participants were also told that they were part of a group of 150 AMT users and that only members of this group who received an invitation could actually participate and receive the promised payment. The instructions further informed participants that the study had allocated a limited number of invitations to be distributed to randomly selected participants, whom we will here call “seeds.” The seeds were invited to participate by the experimenters. In addition to these invitations created by the experimenters, each participant who received and accepted an invitation had the option to create a new invitation and allow one more person than otherwise to participate. However, in order to create a new invitation, the participant had to be willing to donate his or her bonus, even though this would reduce the participant’s earnings which would then be limited to just the base payment. If the participant chose to donate his or her bonus, a recipient of the new invitation (called “invitee”) would then be randomly selected from the other 149 AMT users in the group. The instructions explained further that when a participant donated his or her bonus, we supplemented the bonus amount so that the next invited participant received the same base payment and bonus and had the same options: to keep his or her bonus or donate it and create a new invitation for one more participant.

The instructions were identical for seeds and invitees, with one exception. Unlike those invited by the experimenters (i.e. the seeds), the recipients of participant-generated invitations (i.e. the invitees) were informed that they were given the opportunity to complete the task because another participant had donated his or her bonus (referred to hereafter as “donated invitations”). This one sentence is the only difference in the treatment received by seeds and invitees and provides a very conservative test of the effects of receiving and observing help, given that participants in both treatment conditions received invitations, with the only difference being the source of the invitation and no difference in the size of the bonus that accompanied the invitation. Information is all that was manipulated; there was no difference in the amount of money received.

All participants knew that the person who receives the donated invitation would not know the identity of the participant who made the donation. Thus, anyone receiving a donated invitation was unable to directly reciprocate or to pass along a favorable reputation. We referred to participants by their AMT worker ID, randomly anonymized in a way that precluded the possibility to identify the same individual and be influenced by reputation. We used anonymized identifiers to refer to the other participants in order to dispel any suspicion of deception and to make the information more prominent and compelling. (The detailed instructions used in the study are included in Experiment Instructions S1.).

### Treatments

The experiment involved five manipulations:

- Whether the participant received a donated invitation created by another participant. Some participants were only selected as seeds while others were only selected as invitees. Still other participants were selected as invitees after having been previously selected as seeds. (Previous invitees were ineligible to be selected as seeds since this violated the concept of a seed as the first mover in a sequential decision process.) Invitees were explicitly informed that they were given the opportunity to complete the task because another participant had donated his or her bonus and created the invitation they received.
- The number of times the participant was invited to play the game, either as a seed or invitee. Participants were randomly selected to take part in the game (as a seed or invitee) between one and six times.
- Whether the participant was able to observe donated invitations. In the observation condition, both seeds and invitees were informed about the number of donated invitations that had been created by other participants in their group up to that point in time and saw a list of the pairs of givers and recipients. Participants were permanently assigned to either the observation or no-observation condition; otherwise the effects of observation would carry over to affect behavior in the no-observation condition as well.
- The number of donated invitations the participant observed. Participants in the observation condition were randomly selected to observe different numbers of invitations donated by the members of their group, ranging from zero to 223 observed invitations. Since the number of invitations created by other participants could stay the same or increase, a participant who interacted multiple times in the observation treatment could observe only a higher number of donated invitations in subsequent interactions. Participants could see the total number of donated invitations as well as a list of donors and invitees (with the AMT worker IDs modified to preserve anonymity). Alternatively, we could have displayed the number of members who had chosen to donate, but this would understate members’ level of effort since it would not reflect multiple donations.
- The payment the participant received. Previous research on prosocial behavior has shown that the willingness to donate depends in part on the resources that are available [20]. We manipulated the payment in order to measure the robustness of the results across different incentives to return the bonus. In the high payment treatment, participants received $2 base rate and $1 bonus and in the low payment treatment, they received $1 base rate and $1 bonus. Participants were permanently assigned to either the high or low payment condition.

The two between-individual manipulations, observation: yes/no and payment: high/low were crossed to define four between-individual treatment groups to which participants were randomly assigned. The number of invitations received and observed varied within individual. The number of seeds and invitees varied across treatment groups due to differences across treatments in the rate at which participants were willing to donate (Table S1 in Materials and Methods S1).

## Results

A total of 573 AMT users participated in the experiment, with a mean number of interactions of 2.1 (ranging from 1 to 6), for a total of 1,196 observations. For the analyses, we removed data from 55 participants (126 observations) who required more than five attempts to answer the five multiple-choice questions correctly or whose written summaries revealed an apparent lack of understanding of the instructions. (The results do not change qualitatively if we include participants who required fewer attempts to correctly answer the questions. The results are also qualitatively similar if we use all observations. See Table S2 in Materials and Methods S1.) This left 518 individuals and 1,070 observations, with between 1 and 6 observations per individual (mean of 2.1 and median of 2 observations).

Participants had a mean age of 30.0 (ranging from 17 to 70; Amazon does not allow minors to create and maintain AMT accounts, so the two individuals who reported age under 18 must have either reported incorrect information or used an adult’s AMT account), were 38.8% female, with a median household income of $40,000–49,999. The sample consisted of 91.3% US citizens and 6.0% Indian citizens, the remaining being from other countries. The most common ethnicities were 72.2% White and 13.7% Asian. 29.3% reported being non-religious and 25.5% atheists, while Christianity was the most common religion (10.4% Protestant, 9.9% Roman Catholic, and 12.4% other Christian). 12.9% reported educational attainment of high school or less, 42.3% some college or Associate’s degree, 35.5% Bachelor’s degree, and 9.3% graduate degree. (For detailed demographics of the sample, see Table S3 in Materials and Methods S1.).

In 68.1% of all interactions, participants chose to donate their bonus and thereby create an invitation for a stranger at personal expense (62.0% in the low-payment condition and 74.1% in the high-payment condition). Subjects were also relatively consistent in their behavior – out of the 327 individuals who interacted more than once, only 47 varied their decision.

We used random-intercepts logistic regression models of observations nested in individuals to estimate the change in the odds of donating under the different manipulations. The models allow us to adjust for the non-independence of repeated measures and control for the effect of payment level and two other potential confounders – the time elapsed between subsequent interactions and the number of previous interactions, both of which differed between seeds and invitees since invitees on average interacted with greater frequency compared to seeds. To better isolate the mechanisms, the models pool data only form the relevant treatment conditions: we test GR in the no-observation condition only, we test TPI for seeds only, and we test the interaction of GR and TPI in the observation condition only. We report odds ratios which have a more intuitive interpretation than logistic coefficients. It is important to note that the baseline condition in which participants neither receive nor observe donated invitations does not completely isolate unconditional generosity as a mechanism because returning one’s bonus and creating an invitation slightly increases one’s chance to be invited again and hence, could be strategically motivated. Future research could address this possible confound by manipulating group size, but the focus in the present study is on isolating the effects of GR and TPI, which are not confounded by strategic motivation since the possibility to be re-invited is exactly the same for seeds and invitees.

We tested GR by manipulating whether participants in the no-observation condition were seeds or invitees (H1.1) and also the number of donated invitations they received (H1.2). Few participants received more than two invitations; hence we binned these as two or more. The results are limited to the no-observation condition (*N* = 516) to avoid confounding the effects of receiving and observing invitations (since the more invitations that other participants have previously sent, the higher the number of invitations that can be observed as well as received).

**Figure 3:**
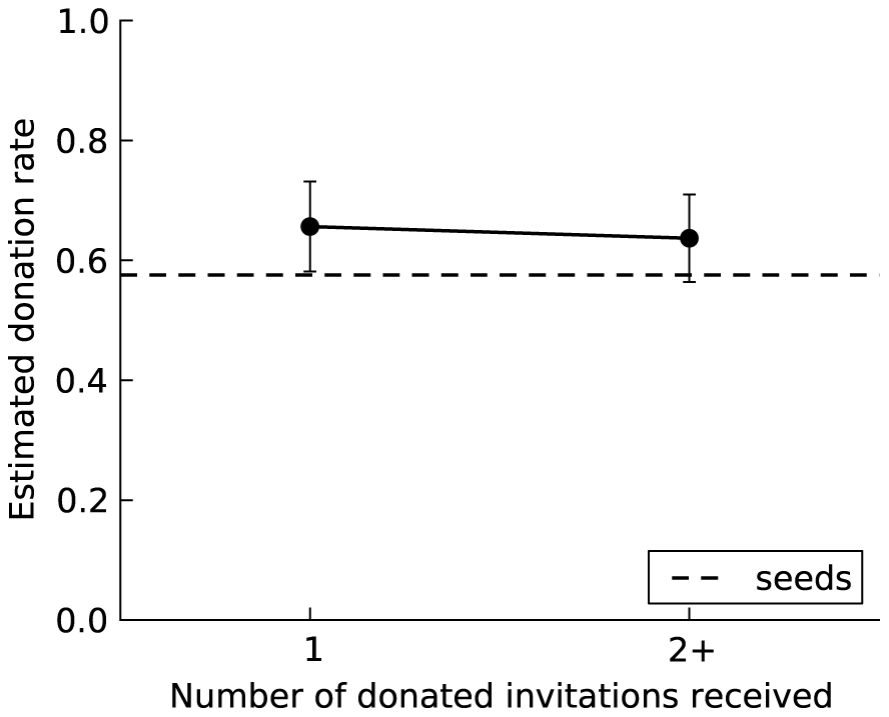
The effect of generalized reciprocity on the willingness to donate in the no-observation condition. To facilitate interpretation of the odds ratios, the figure shows the estimated donation rate and 95% confidence intervals based on a random-intercept linear regression model with robust standard errors corresponding to the random-intercept logistic model in Table 1A. The robust standard errors adjust for possible heteroskedasticity with a binary dependent measure. The dashed line shows the baseline donation rate among seeds in the no-observation condition. The donation rate is significantly higher among invitees than among seeds after receiving one donated invitation but does not continue to increase with receipt of additional invitations.

Consistent with GR, Table 1A reveals a seven-fold increase in the odds of donating (*p* = 0.030) among invitees compared to the baseline odds for seeds. Although statistically significant, the change in behavior was relatively small, as evident in Figure 3, which reports the change in the fraction donating (rather than the odds), and only within individuals (Table S5 in Materials and Methods S1). This small effect size may reflect the minimal GR stimulus, which consisted of a single short statement informing invitees that their invitation was created by another participant who had donated his or her bonus to make that possible.

**Table 1:** Odds Ratios for Donating Across Treatments.

Manipulation		A) GR	B) TPI+	C) TPI–	D) GR × TPI
Invitee (receives a donated invitation)		7.006*			0.327
		(0.030)			(0.262)
Has previously received donated invitations		0.712			1.021
		(0.686)			(0.982)
Seeds					
	Observes 0–75		11.414*	(baseline)	(baseline)
			(0.043)		
	Observes 76–150		1.341	0.047	0.136
			(0.787)	(0.215)	(0.101)
	Observes 151+		0.219	0.003	0.015*
			(0.280)	(0.198)	(0.022)
Invitees					
	Observes 0–75				(baseline)
				
	Observes 76–150				19.907*
					(0.041)
	Observes 151+				89.948*
					(0.026)
High payment		64.103**	2.532	0.858	3.235
		(0.007)	(0.300)	(0.930)	(0.295)
Time waited (in hours)		0.972*	0.992	1.019	0.976
		(0.023)	(0.577)	(0.619)	(0.075)
Previous participations		0.690	0.784	1.347	0.454
		(0.379)	(0.622)	(0.848)	(0.171)
Baseline odds		4.305	5.323	152.785	268.707***
		(0.181)	(0.100)	(0.130)	(0.000)
Number of observations		516	371	175	554
Number of participants		252	277	133	266
Wald 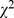		5 df, 11.93*	6 df, 6.66	5 df, 2.49	8 df, 11.98
		(0.036)	(0.354)	(0.778)	(0.214)

Two-sided tests: *p<0.05, **p<0.01, ***p<0.001.

The table reports odds ratios and p values (in brackets) from random-intercept logistic regression models for A) seeds and invitees in the no-observation treatment by number of donated invitations received; B) seeds in the observation and no-observation treatments by number of donated invitations observed; C) seeds in the observation treatment by number of donated invitations observed; and D) seeds and invitees in the observation treatment by number of donated invitations observed by invitees compared to seeds. Results show that receiving and observing donations initially increases the willingness to help others, and that invitees are less susceptible to a subsequent decline in helping.

In sum, participants were more likely to be generous towards a stranger after experiencing generosity. However, the effect is limited to the first receipt of generosity as the critical event in triggering GR. The odds of donating do not continue to increase but instead slightly decrease with receiving additional donated invitations. A plausible explanation is that participants may feel they fulfilled their normative obligation to “pay it forward” when they donated their bonus after their first donated invitation.

**Figure 4:**
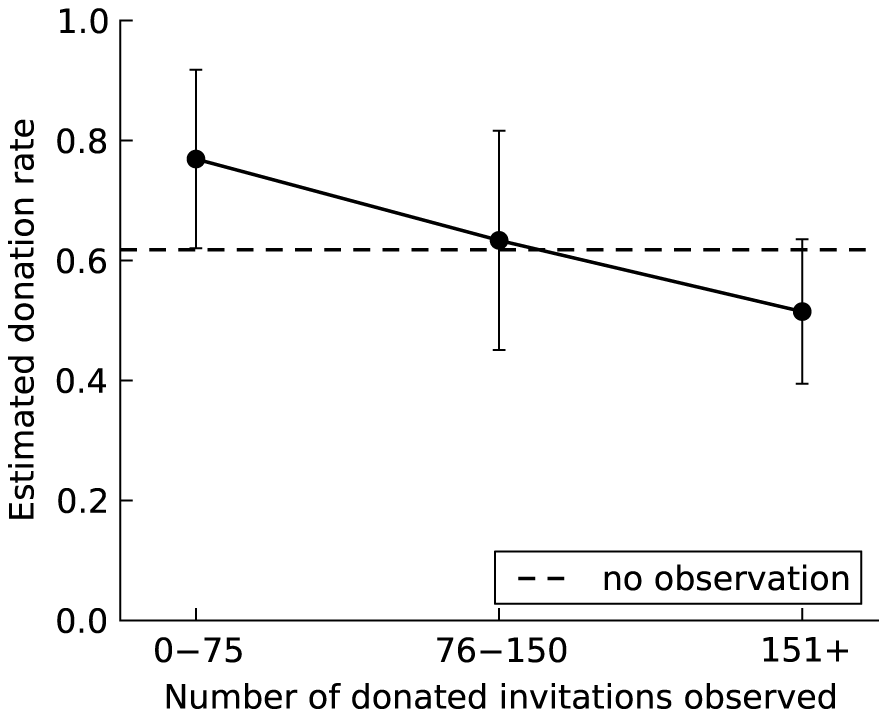
The effect of third-party influence on the willingness to donate among seeds. To facilitate interpretation of the odds ratios, the figure shows the estimated donation rate and 95% confidence intervals based on a random-intercept linear regression model with robust standard errors corresponding to the random-intercept logistic model in Table 1B. The robust standard errors adjust for possible heteroskedasticity with a binary dependent measure. The dashed line shows the baseline donation rate among seeds in the no-observation condition. The donation rate is significantly higher after observing 0–75 donations by other group members but then declines as the level of observed donation increases further. However, the decline is within the confidence intervals of the estimated donation rates, consistent with the results in Table 1C (in which the donation rates are compared across levels of observed donation).

We tested TPI by manipulating whether participants observed invitations created by others and the number of donated invitations they observed. Due to the sparsity of data with 223 levels of observed donation and 266 participants, we binned the number of observed donated invitations into three levels: 0–75 (up to about one-third the total number of donations), 76–150 (between one-third and two-thirds), and 151+ (more than two-thirds). Consistent with the expected effects of TPI, Table 1B and Figure 4 show a statistically significant increase in the odds of donating (OR = 11.41, *p = *0.043) among the seeds who had observed between 0 and 75 donated invitations, compared to those who had not observed any. However, the level of donation among those who observed more than 75 invitations was not significantly greater than the baseline level.

This is also consistent with the results for a model that directly tests for changes in the level of donation among seeds as the number of observed donations increases (reported in Table 1C). Here the baseline is lowest level of observed donation instead of the no-observation condition. Although the direction of the effect is as predicted, the decrease in the probability of donation as the number of observed donations increases is not statistically significant (OR = 0.047, *p = *0.215 for observing 76–150; OR = 0.003, *p* = 0.198 for observing 151+; 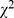 (1 df) = 1.08, *p* = 0.298 for the difference between observing 76–150 and observing 151+). Similarly to GR, the effect of TPI appears to be non-linear, with most of the effect evident at relatively low levels of observed donation and little subsequent change. The conclusion does not change with more fine-grained categories. The rate of donation decreases (albeit not significantly) as the number of observed donations increases from 0–25 to 26–50 to 51–75.

**Figure 5:**
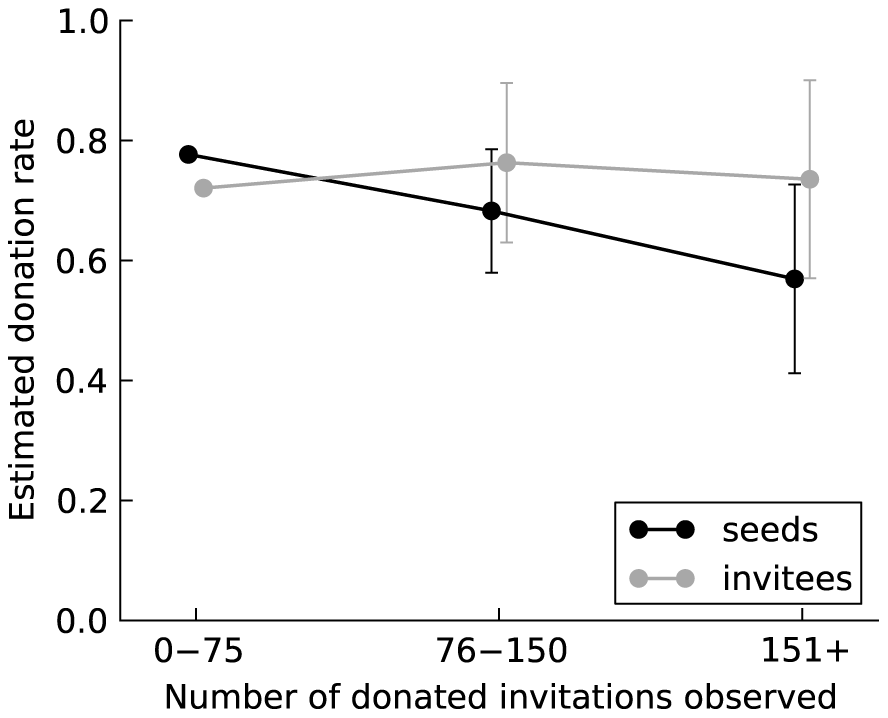
The effect of third-party influence on the willingness to donate among seeds and invitees. To facilitate interpretation of the odds ratios, the figure shows the estimated donation rate and 95% confidence intervals based on a random-intercept linear regression model with robust standard errors corresponding to the random-intercept logistic model in Table 1D. The robust standard errors adjust for possible heteroskedasticity with a binary dependent measure. Relative to the 0–75 baseline, the donation rate declines with the level of observed donation among seeds but not among invitees.

However, as the theory of GR suggests, the effect from observing widespread generosity is significantly different for those who have recently benefited from generosity compared to those who have not. When observing more than 75 donated invitations, the odds of donating decrease for seeds but do not change for invitees (Table 1D and Figure 5). This difference in the odds-ratios between seeds and invitees is statistically significant (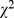 (1 df) = 3.88, *p* = 0.049 for observing 76–150; 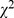 (1 df) = 5.55, *p* = 0.019 for observing 151+) and suggests the possibility that seeds eventually succumb to a “bystander” (or “free-rider”) effect from which invitees are immune due to having been recipients of generosity. This apparent immunity suggests that an injunctive norm to “pay it forward” does not diminish when the level of helping behavior is high, while a descriptive norm to “be generous if that is what others are doing” is less resistant to the temptation to “let George do it” as the opportunity to do so increases.

Finally, our analyses also show that the odds of donating are larger in the high-payment condition, especially among seeds in the no-observation condition, as shown in Table 1A. Nevertheless, the effects of GR and TPI do not significantly vary by payment (Table S6 and Table S7 in Materials and Methods S1). There was no significant change in the odds of donating with the wait time between invitations or with the number of times one has previously interacted. (We also tested the effect of demographic variables on the odds of donating and apart from a positive effect from age, demographics do not affect generosity, as reported in Table S4 in Materials and Methods S1.).

## Discussion

Social contagion offers a compelling theoretical explanation for the emergence and spread of generous behavior, especially when directed towards strangers or in large groups where there is a very low probability that generosity will be directly reciprocated. This study investigated two mechanisms that might explain the contagion of generosity – generalized reciprocity and third-party influence. Causal mechanisms are notoriously difficult to observe in natural settings, and controlled diffusion experiments with large groups are highly impractical in traditional laboratory settings. We therefore designed and conducted a large behavioral experiment online. The experiment used anonymity to isolate the effects of the contagion mechanisms from other cooperation-inducing mechanisms, including direct and indirect reciprocity, as well as peer pressure based on reputation effects. The experiment disentangled the effects of receiving and observing generous behavior by manipulating whether participants benefited from the willingness of others to donate their bonus payment, the number of times they benefited, whether participants were informed of the extent of third party donations, and the number of donations they observed. To ensure the robustness of the results across different incentive levels, we also manipulated participants’ payments.

The experimental results show that receiving and observing generosity can significantly increase the likelihood to be generous towards a stranger. However, the results are also consistent with the “bystander” hypothesis that the willingness to contribute can be offset by lower perceived need when the level of helping is sufficiently high. This bystander effect is especially evident among those who have not themselves benefited from generosity, suggesting an important difference between injunctive and descriptive norms: once the level of generosity is sufficient to establish a descriptive norm to be generous towards others, further increases in the level of generosity do not strengthen the norm but instead signal that one’s own contribution is not needed. However, an injunctive norm to reciprocate generosity by “paying it forward” does not appear to depend on the belief that one’s own contribution is needed. Framed by Cialdini’s extensive research [14], it seems that the need for help alone is not sufficient to motivate generous behavior unless coupled with either an injunctive or descriptive norm, and norms are not sufficient unless coupled with the need for help, especially if the norm is descriptive.

The study contributes to knowledge about prosocial behavior, altruism, and reciprocity by adopting a relational perspective in a research line that has generally focused on individuals responding independently or in aggregates but rarely as nodes of a social network. We also contribute to knowledge about social contagion by investigating the interaction between transmission through direct contact and transmission through third-party influence, two mechanisms that have been usually studied independently in previous contagion research. We advance social science methodology by developing, demonstrating, and evaluating an online platform for studying the diffusion of behavior in large social groups under controlled conditions, something that is not feasible in a traditional laboratory setting.

In addition to a greater insight into the theoretical puzzle of generosity toward strangers (in the absence of clear opportunities for personal gain), the possibility that generous behavior can trigger cascades has important practical applications, including fund-raising efforts for public broadcasting, contributions to online collaborative projects, and creative participation in online content communities. Our empirical findings could inform strategies for more effectively targeting and structuring interventions intended to promote pro-social behavior, generosity, and cooperative ventures in large groups and organizations, with potential use by philanthropists, activists, policy makers, managers, and administrators.

However, it is important to note that although GR and TPI may be able to increase the level of generosity in a community, they may not be sufficient to jump start the emergence of cooperation. In particular, GR has been shown to be unstable as a strategy for the evolution of cooperation [43]. Rather, GR is a behavioral pattern that coevolved with cooperation mechanisms such as direct reciprocity, indirect reciprocity, group selection, and spatial structure [25], [44].

Although the experimental design helps disentangle the effects of GR and TPI, a word of caution is in order. While the AMT participants are much more diverse than the college students used in most previous experiments on prosocial behavior, the sample is nevertheless not perfectly representative of the general population. Future research should replicate the study with other populations with different demographic profiles in order to test whether the findings can be generalized to other populations. Ideally, the external validity of the study should be confirmed in a field experiment with stronger manipulations and more meaningful donations. Such field experiment will be also better suited than the online experiment we conducted to gauge the size of the GR and TPI effects and the practicality of possible interventions. Future research could also extend the present study by testing whether egoistic behavior (e.g. stealing or free-riding) can also spread as an “anti-social” contagion, through influence (TPI) or “generalized retaliation” (GR). The online experimental platform that we developed for the current project can be improved and easily adapted to study other populations, with different stimuli, and with participants embedded in large social networks.

Another promising direction for further research is to investigate the macro-level effects of GR and TPI. The effects of GR are limited to the one person who is helped, while the effects of TPI can extend to large numbers of people who observe helping behavior. Thus, TPI may be vital in the early stages of a contagion, by multiplying the number of cascades, while GR could be more beneficial in the later stages, by reinforcing a widely held descriptive norm with an emergent injunctive norm. This reinforcement may be essential in offsetting the growing belief that one’s own efforts are not needed as more people are observed to help others. Moreover, these dynamics may depend as well on the structure of social networks that limit the horizons for the observation of helping behavior. The implications of network structure for the dynamics of helping cascades driven by GR and TPI are not intuitively obvious, and we expect agent-based models may prove helpful in generating new hypotheses that can then be tested in a new line of research using online experiments.

## Supporting Information

Experiment Instructions S1 **Recruitment posting, e-mail invitation, and consequent screens from the experiment website.**
(PDF)

Materials and Methods S1 **Supporting information on experimental procedure and analyses, containing Tables S1**–**S7.** Table S1, Number of observations and number of participants by experimental manipulation. Table S2, Odds ratios for donating across treatments for the complete sample. Table S3, Detailed demographics for the participant sample. Table S4, Odds ratios for donating as predicted by demographic variables. Table S5, Odds ratios for donating across treatments with disaggregated between-individual and within-individual effects. Table S6, Odds ratios for donating across treatments for the low payment condition. Table S7, Odds ratios for donating across treatments for the high payment condition.
(PDF)
