# Supplementary material for: The Social Contagion of Generosity
Source: PLoS One. 2014 Feb 13;9(2):e87275. doi: 10.1371/journal.pone.0087275 (PMC3923723; doi:10.1371/journal.pone.0087275)
Supplement: Experiment Instructions S1 — Recruitment posting, e-mail invitation, and consequent screens from the experiment website. (PDF) [file pone.0087275.s001.pdf]

Experiment Instructions S1

Tsvetkova and Macy, *The Social Contagion of Generosity*

1. Recruitment posting

Please read the instructions carefully. Your attention will be tested.

Sign up to participate in the Invitation Game

You are invited to sign up for the chance to participate in a research study on decision making called "The Invitation Game." You can participate in the study only after receiving an e-mail invitation from us with instructions. The study will take place over the next two weeks. Over the course of the study, you may receive multiple such invitations. Each time you receive an invitation, you will have 24 hours to complete the same simple task. This task requires you to demonstrate understanding of the game rules and to take a single decision. **The task takes less than 10 minutes to complete and pays $1 base rate and, depending on your decision, $1 bonus. Since there is the possibility to do the task multiple times, you may eventually earn up to 7 * $2 = $14!**

If you agree to sign for the study by completing and submitting this HIT, you will enter a group of potential participants. Not everyone in this group will receive invitations. Which turkers receive invitations will be determined by a lottery. The selection will be completely at random. This means that your answers to the questions below or later in the game will not be used in the selection. Further, this means that **you will be eligible to be selected to receive an invitation multiple times, but there is no guarantee that you will be selected even once.**

If you have been selected to receive an invitation, you will receive an e-mail with instructions at the e-mail address associated with your MTurk worker account. PLEASE MAKE SURE THAT YOU CHECK YOUR E-MAIL EVERY DAY OVER THE NEXT TWO WEEKS! If you do not respond to an invitation within 24 hours, we will assume that you are no longer interested in participating and we will remove you from the pool of potential participants. Further, you cannot be selected for a new invitation if you currently have an active, unanswered invitation. Hence, you should try and respond to an invitation as soon as possible.

Taking part in the study is completely voluntary. If you decide to take part and sign up now, you are free to withdraw at any time.

We do not anticipate any risks to you participating in the study other than those encountered in everyday use of the Internet. Your answers will be confidential. The records of this study will be

kept private; only the researchers will have access to the records. In any sort of report we make public we will not include any information that will make it possible to identify you.

To confirm that you have read the above information and that you consent to take part in the study, please type the text *I agree* **here:** . (*Please note that we include this step to screen out turkers who do not read instructions carefully. <u>You will not be paid if you did not type the correct text!</u>*)

To guarantee that you will not discuss the study with other turkers (in person or on forums), please check this box: . (*Please note that we include this step to ensure that we have independent and unbiased responses. <u>You will be paid but not allowed to participate in the study if you did not check the box!</u>*)

In addition, please answer a short standard survey. Your answers to the questions below have no relevance as to whether you are selected to receive an invitation for the game task. We will only use this information once we have collected all results and completed the study. Hence, please answer truthfully. (*If you do not want to answer a particular question, please do not select/write an answer.*)

What is your gender?

What is your age?

What is the highest level of education you have completed?

What is the total income of your household?

What is your religious affiliation?

What is your ethnicity?

What is your nationality?

2. E-mail invitation

From: Cornell SDL <mvt9@cornell.edu>
Subject: You have been invited to the Invitation Game

Message from Cornell SDL (mvt9@cornell.edu)

Dear turker,

You have been invited to complete the task associated with the MTurk HIT "Sign up to participate in the Invitation Game," which you submitted. Your invitation is valid for the next 24 hours.

To complete the task, please use the following information:

* MTurk Worker ID: A27L6Z6PBCE04Y
* Invitation ID: ILUS

and:

1. Go to https://sdlab.soc.cornell.edu/study11/igame/ and complete the task.
2. After you have completed the task, go to https://www.mturk.com/mturk/preview?groupId=2FH56XBAT2D5PP8RRYUQ7JG7YZP04I and submit the HIT.

Thank you for your participation!

Best regards,

Milena Tsvetkova, Cornell SDL

***
If you have questions, you may contact me at mvt9@cornell.edu. If you have any questions or concerns regarding your rights as a subject in this study, you may contact the Cornell Institutional Review Board (IRB) at 607-255-5138 or access their website at http://www.irb.cornell.edu. You may also report your concerns or complaints anonymously through Ethicspoint (www.hotline.cornell.edu) or by calling toll free at 1-866-293-3077. Ethicspoint is an independent organization that serves as a liaison between the University and the person bringing the complaint so that anonymity can be ensured.

Greetings from Amazon Mechanical Turk,

The message above was sent by an Amazon Mechanical Turk user.
Please review the message and respond to it as you see fit.

Sincerely,
Amazon Mechanical Turk
https://workersandbox.mturk.com
410 Terry Avenue North
SEATTLE, WA 98109-5210 USA

3. Consecutive screens from the experiment website

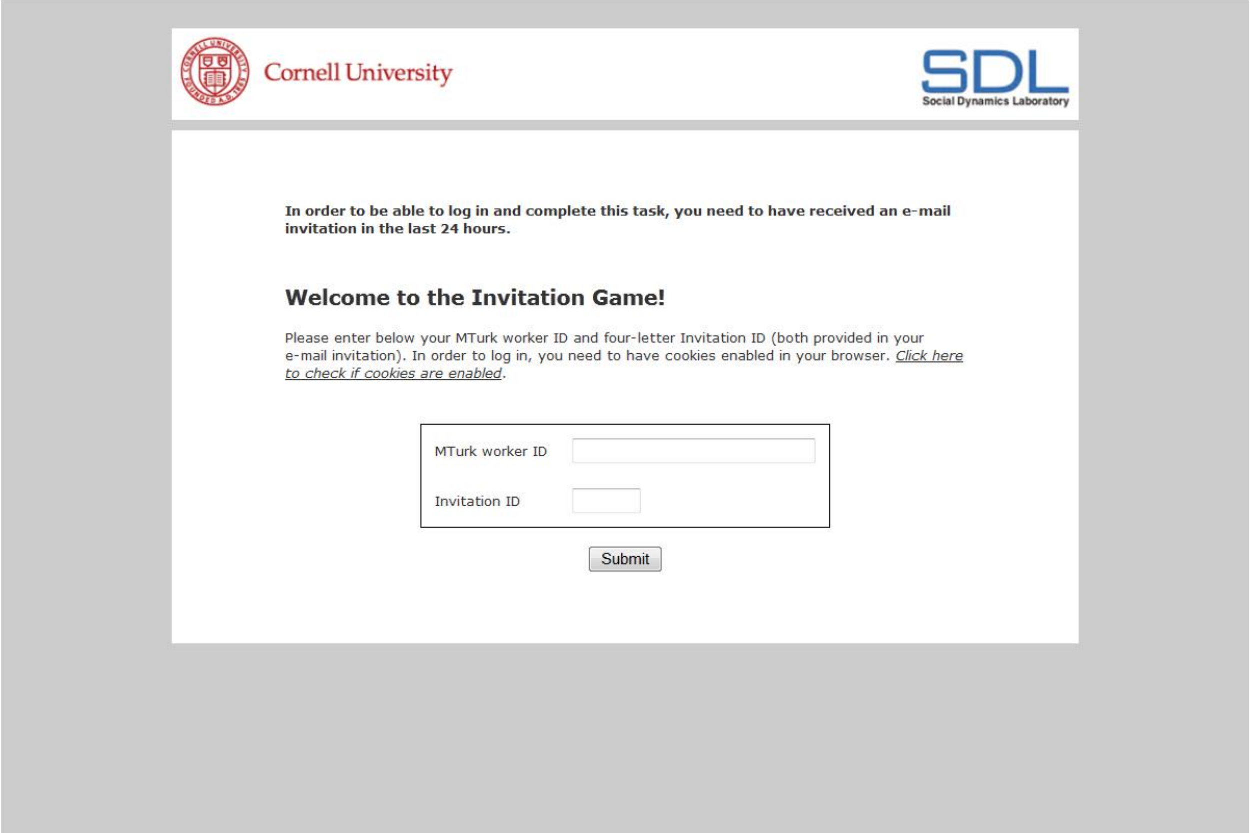

Cornell University

SDL
Social Dynamics Laboratory

Since the amount of money you receive in the game depends on the decisions that you and the other participants make, it is important that you understand and remember the game rules well. Please read the instructions below carefully and then answer five questions about them. You will need to demonstrate your understanding of the rules before you can proceed to make your decision.

How the Invitation Game Works

You are part of a group of 150 turkers who have signed up to participate in the game. To participate in the game, you need an invitation. Unfortunately, we do not have invitations available for everyone and hence, we send invitations to randomly selected participants. Each participant who receives and accepts an invitation receives a payment of $1 and a bonus of $1. However, they then have the choice to return their bonus, in which case we create a new invitation and allow one more person than otherwise to participate. The recipient of the new invitation is randomly selected from the other 149 turkers in the group. This means that a participant is not eligible for the invitation they create but otherwise, they may be selected to receive any invitation created by any of the other 149 participants in the group.

When a participant returns their bonus, we multiply the bonus amount by 2 and thus, the next invited participant receives the same $1 regular payment and $1 bonus. This next participant also faces the same choice: they need to decide whether to keep their bonus or return it and create a new invitation for one more turker. This means that if participant A decides to return their bonus and invite turker B, and then turker B also decides to return their bonus, the chance that participant A is selected to complete the task again increases. If participant A decides to keep their bonus, we will not create and send an invitation to turker B. However, regardless of participant A's decision, participant A remains eligible for any invitations currently outstanding or created in the future by others in the group.

The identities of all participants will be kept anonymous and the decisions made by individual participants will not be revealed to others.

Now, please answer the following questions. You will not be allowed to proceed until you have answered all questions correctly.

The game starts with a small number of invitations allocated by the researchers but recipients of invitations may create new invitations. ○ True ○ False

In order to receive an invitation,

- ○ one needs to have answered all questions correctly.
- ○ one needs to have already created many invitations.
- ○ one needs to be randomly selected from the members of one's group.

Every invited participant receives the same payment and bonus and faces the same choice options regarding what to do with their bonus. ○ True ○ False

As a participant, if you decide to return your bonus and create a new invitation,

- ○ you may be selected to receive the invitation you create.
- ○ you cannot receive the invitation you create but you may be selected to receive other invitations, including the invitation created by the recipient of your invitation.

As a participant, if you decide to keep your bonus,

- ○ you may still be selected to receive invitations.
- ○ you exit the game and can no longer receive any invitations.

Submit

(Screen for seed in the no-observation, low-payment treatment group.)

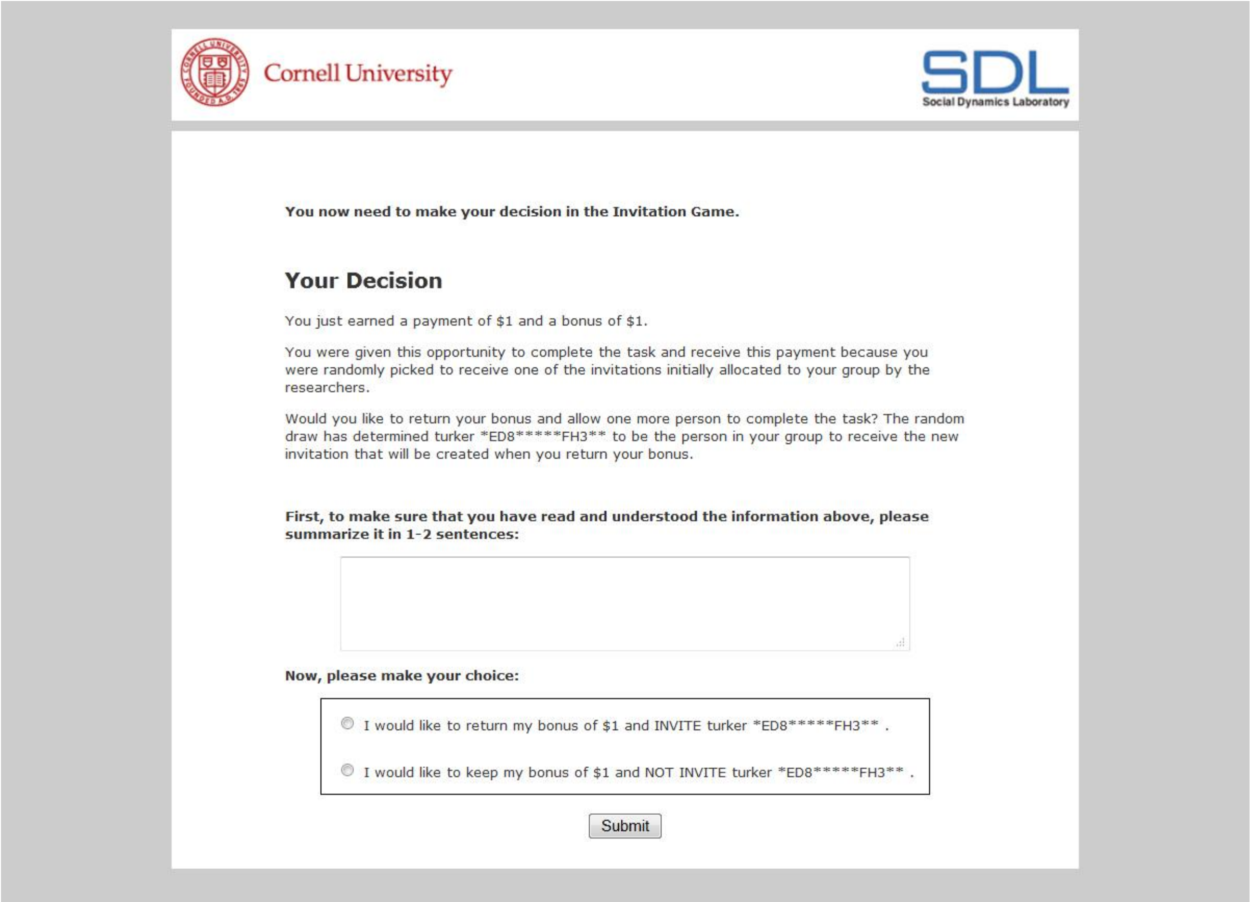

Cornell University

SDL Social Dynamics Laboratory

You now need to make your decision in the Invitation Game.

Your Decision

You just earned a payment of $1 and a bonus of $1.

You were given this opportunity to complete the task and receive this payment because you were randomly picked to receive one of the invitations initially allocated to your group by the researchers.

Would you like to return your bonus and allow one more person to complete the task? The random draw has determined turker *ED8*****FH3** to be the person in your group to receive the new invitation that will be created when you return your bonus.

First, to make sure that you have read and understood the information above, please summarize it in 1-2 sentences:

Now, please make your choice:

- I would like to return my bonus of $1 and INVITE turker *ED8*****FH3** .
- I would like to keep my bonus of $1 and NOT INVITE turker *ED8*****FH3** .

Submit

(Screen for invitee in the observation, low-payment treatment group.)

Cornell University

SDL
Social Dynamics Laboratory

You now need to make your decision in the Invitation Game.

Your Decision

You just earned a payment of $1 and a bonus of $1.

You were given this opportunity to complete the task and receive this payment because turker E****W**J*3*NK invited you. That is, turker E****W**J*3*NK elected to return their bonus of $1 and create a new invitation. The person who was randomly picked to receive the new invitation was you.

So far, in your group, there have been 3 other cases in which a turker decided to return their bonus and as a result, there have been 3 other new invitations created:

Turker *ED**JF*F***N* invited turker ***3*3*J**F*W1
Turker *E*3*3*J*K**W* invited turker **8*E*JFH**F**
Turker 23*J***F****OJ invited turker **WKO**3JD****

Would you like to return your bonus and allow one more person to complete the task? The random draw has determined turker *3***S**JFKO** to be the person in your group to receive the new invitation that will be created when you return your bonus.

First, to make sure that you have read and understood the information above, please summarize it in 1-2 sentences:

Now, please make your choice:

- I would like to return my bonus of $1 and INVITE turker *3***S**JFKO** .
- I would like to keep my bonus of $1 and NOT INVITE turker *3***S**JFKO** .

Submit

Cornell University

SDL
Social Dynamics Laboratory

You are now finished with the task.

Thank you!

Since you chose to invite another turker to complete the task, you will not receive a bonus. The invitation to turker HEU**3****FI** has just been sent.

To obtain your payment of $1, please go to the MTurk site and submit the HIT. You will need your Invitation ID, so please copy it now: **QNNX**.

Do not forget that you may be invited to participate again, so please check your e-mail regularly!

Take me to the MTurk site to submit the HIT.
