# Supplementary material for: The Social Contagion of Generosity
Source: PLoS One. 2014 Feb 13;9(2):e87275. doi: 10.1371/journal.pone.0087275 (PMC3923723; doi:10.1371/journal.pone.0087275)
Supplement: Materials and Methods S1 — Supporting information on experimental procedure and analyses, containing Tables S1–S7. Table S1, Number of observations and number of participants by experimental manipulation. Table S2, Odds ratios for donating across treatments for the complete sample. Table S3, Detailed demographics for the participant sample. Table S4, Odds ratios for donating as predicted by demographic variables. Table S5, Odds ratios for donating across treatments with disaggregated between-individual and within-individual effects. Table S6, Odds ratios for donating across treatments for the low payment condition. Table S7, Odds ratios for donating across treatments for the high payment condition. (PDF) [file pone.0087275.s002.pdf]

# Materials and Methods S1

Tsvetkova and Macy, *The Social Contagion of Generosity*

## 1. Experimental procedure

Experiments under controlled conditions are the best method available to investigate causal processes. In particular, laboratory experiments have become established as the mainstream method for studying behavioral mechanisms. However, studying social contagion requires large groups to observe the occurrence of cascades and gathering a large number of participants over extended periods of time in a laboratory poses a challenge. We resolved this problem by conducting an experiment with human subjects interacting online.

The study was conducted over a period of six weeks in March-April, 2013, in two sessions, each lasting 10-14 days. The two sessions corresponded to the two different payment treatments. To avoid learning effects, we did not allow AMT users who participated in the first session to sign up for and participate in the second session. Further, we scheduled the two sessions two weeks apart in order to minimize carry-over effects due to participants obtaining a lower base rate than the rate they might remember from the recruitment advertisement for the previous session.

Since recipients of invitations were randomly selected, not all of the AMT users assigned to the four 150-person groups received an invitation. Further, not all of the AMT users who received invitations responded to them. 662 individuals received at least a first invitation, to which 89 did not respond, either because they did not check their e-mail on time, they did not have an opportunity to respond on time, or they were no longer interested in participating. If a

participant did not respond to an invitation within 24 hours, we removed that participant from the group, added another randomly selected AMT user from the participant pool to the group to maintain 150 members, and forwarded the unanswered invitation to another randomly selected group member.

**Table S1. Number of observations and number of participants (in brackets) by experimental manipulation.** The brackets for seeds and invitees show the number of unique participants who interacted only as seeds or only as invitees. The brackets in the "Total" column count also the participants who interacted as both seeds and invitees.

| | | Seed | Invitee | Total |
|---|---|---|---|---|
| No observation | Low payment | 112 (40) | 136 (47) | 248 (126) |
| | High payment | 84 (26) | 184 (61) | 268 (126) |
| Observation | Low payment | 93 (29) | 185 (65) | 278 (129) |
| | High payment | 82 (27) | 194 (68) | 276 (137) |
| Total | | 371 (122) | 699 (241) | 1070 (518) |

The experiment did not involve deception of any kind. Invitations were actually created by participants. Hence, the number of donated invitations participants received or observed depended on the number of previous participants who had chosen to donate their bonus. Thus, avoiding deception came at the cost of endogenizing these manipulations. However, we took concrete measures to reduce any confounding effects from the endogenous manipulations. First, we invited new seeds throughout the experiment in order to minimize the difference in waiting time for first invitation between seeds and invitees. On average, seeds received their first invitation 49 hours after signing up for the study (min = 0.8, max = 130); for invitees, the average waiting time was about 56.5 hours (min = 0.4, max = 198). Second, the analyses control for the time between interactions (for the first interaction, this is the time elapsed since signing

up for the study) to account for any remaining difference in waiting time and for the fact that invitees interacted more often than seeds due to the high level of generosity. Third, we did not inform participants when the experiment in their group started, how many seed invitations had been already sent out, and when the experiment in their group was to end (participants only knew that they may be selected to participate anywhere between 0 and 7 times). This means that participants did not know what their chances were for receiving another invitation and hence, could not condition their behavior on such knowledge. Similarly, in the observation manipulation, participants did not know what the number of already created invitations implied for the number of future invitations. Since the effect of observation starts decreasing as early as 75 invitations, we do not believe that the non-monotonicity of TPI is driven by an "end-game effect."

## 2. Data

The experimental software (implemented in Python and Django), the data, and the scripts for the analyses are available from the authors upon request. Please direct correspondence to Milena Tsvetkova at mvt9@cornell.edu.

## 3. Internal validity

To improve the internal validity of the study, we required participants to answer correctly five multiple-choice questions that tested their comprehension of the game rules before they could proceed. The questions emphasized that invitations were distributed randomly and that while inviting someone else could increase one's chance to be invited again, not inviting does not

decrease it. In addition to the multiple-choice questions, participants were required to write a short summary to demonstrate that they understood the decision they were asked to make. (See Experiment Instructions S1.)

On average, participants took 1.7 attempts to answer the five questions correctly but the distribution is extremely skewed to the left, with 35 participants who took more than 5 attempts and a maximum of 34 attempts. Participants who required a large number of attempts were likely randomly guessing the answers to the questions without having read or understood the instructions. The summaries written by participants were blindly hand-coded without knowledge of the participant's treatment or decision. Common errors included assuming that the participant exits the game if they do not return their bonus, that the turkers from the list of previous donations or that all other 149 group members will receive invitations if the participant returns their bonus, or that the participant was invited by another turker when in fact they were treated as a seed.

To improve the internal validity of the results, the analyses in Table 1 exclude data from the 55 participants (126 observations) who required more than five attempts to answer the five multiple-choice questions correctly or whose written summaries revealed an apparent lack of understanding of the instructions. In Table S3, we have replicated the analyses for the complete data. The results are qualitatively the same. The major difference is that the effect from GR in A) is smaller and loses statistical significance. The GR treatment was less visible (a 4-line paragraph) compared to the observation treatment (a long list of donors and recipients) and hence, it was probably overlooked by participants who were not paying attention.

**Table S2. Odds ratios for donating across treatments for the complete sample.** Replication of Table 1 for all 1,196 observations and 573 individuals, including individuals who demonstrated poor understanding of the game rules.

| Manipulation | **A) GR** | **B) TPI+** | **C) TPI–** | **D) GR x TPI** |
|---|---|---|---|---|
| Invitee (receives a donated invitation) | 1.931<br>(0.269) | | | 0.283<br>(0.151) |
| Has previously received donated invitations | 0.427<br>(0.207) | | | 1.704<br>(0.515) |
| Seeds | | | | |
| Observes 0-75 | | 7.933*<br>(0.041) | (baseline) | (baseline) |
| Observes 76-150 | | 1.310<br>(0.779) | 0.098<br>(0.243) | 0.164<br>(0.099) |
| Observes 151+ | | 0.221<br>(0.237) | 0.009<br>(0.199) | 0.023*<br>(0.020) |
| Invitees | | | | |
| Observes 0-75 | | | | (baseline) |
| Observes 76-150 | | | | 13.691*<br>(0.043) |
| Observes 151+ | | | | 45.937*<br>(0.028) |
| High payment | 25.920**<br>(0.002) | 2.805<br>(0.194) | 1.012<br>(0.994) | 3.114<br>(0.240) |
| Time waited (in hours) | 0.981<br>(0.052) | 0.992<br>(0.501) | 0.998<br>(0.933) | 0.975<br>(0.042) |
| Previous participations | 1.184<br>(0.599) | 1.150<br>(0.743) | 0.773<br>(0.842) | 0.531<br>(0.198) |
| Baseline odds | 3.023<br>(0.165) | 4.002<br>(0.104) | 196.341<br>(0.092) | 163.839***<br>(0.000) |
| Number of observations | 569 | 415 | 195 | 627 |
| Number of participants | 278 | 310 | 149 | 295 |
| Wald $\chi^2$ | 5 df, 14.03*<br>(0.016) | 6 df, 6.75<br>(0.345) | 5 df, 2.62<br>(0.758) | 9 df, 13.41<br>(0.145) |

Two-sided tests: * $p < 0.05$, ** $p < 0.01$, *** $p < 0.001$

The table reports odds ratios and *p* values (in brackets) from random-intercept logistic regression models for A) seeds and invitees in the no-observation treatment by number of donated invitations received; B) seeds in the observation and no-observation treatments by number of donated invitations observed; C) seeds in the observation treatment by number of donated invitations observed; and D) seeds and invitees in the observation treatment by number of donated invitations observed by invitees compared to seeds.

## 4. Demographics

### Table S3. Detailed demographics for the participant sample (*N*=518)

| Characteristic | Mean/ Percent |
|---|---|
| Female …..………………………………………. | 38.77 |
| Age………………………………………………. | 29.99 (SD=9.56) |
| Income | |
| Less than $10,000…………………………. | 9.67 |
| $10,000 - $19,999…………………………. | 9.86 |
| $20,000 - $29,999…………………………. | 13.73 |
| $30,000 - $39,999…………………………. | 13.73 |
| $40,000 - $49,999…………………………. | 11.22 |
| $50,000 - $59,999…………………………. | 10.83 |
| $60,000 - $69,999…………………………. | 6.77 |
| $70,000 - $79,999…………………………. | 6.58 |
| $80,000 - $89,999…………………………. | 5.80 |
| $90,000 - $99,999…………………………. | 2.90 |
| $100,000 - $149,999………………………. | 7.54 |
| $150,000 or More………………………….. | 1.35 |
| Education | |
| Less than High School…………………….. | 0.77 |
| High School or GED………………………. | 12.16 |
| Some College……………………………… | 34.75 |
| Associate's Degree………………………… | 7.53 |
| Bachelor's Degree…………………………. | 35.52 |
| Graduate Degree (Master's, Doctorate, etc.) | 9.27 |
| Nationality | |
| United States……………………………… | 91.31 |
| India………………………………………. | 5.98 |
| Other………………………………………. | 2.71 |
| Ethnicity | |
| White, non-Hispanic……………………… | 72.15 |
| Asian-Pacific Islander……………………. | 13.73 |
| African-American………………………… | 5.80 |
| Hispanic…………………………………..... | 3.87 |
| Native American………………………….. | 1.35 |
| Other………………………………………. | 3.09 |
| Religion | |
| Non-religious…………………………….... | 29.34 |
| Atheist…………………………….............. | 25.48 |
| Protestant……………………………......... | 10.42 |
| Roman Catholic………………………….. | 9.85 |
| Other Christian…………………………… | 12.36 |
| Hindu……………………………............... | 5.79 |
| Buddhist……………………………........... | 1.74 |
| Jewish…………………………….............. | 1.16 |
| Muslim……………………………............ | 0.77 |
| Other non-Christian……………………… | 3.09 |

**Table S4. Odds ratios for donating as predicted by demographic variables.** The baseline odds are for a thirty-year-old white American Christian male with high-school education or less and household income of less than $10,000.

| Demographics | Odds ratio ($p$ value) |
|---|---|
| Age | 1.125** (0.007) |
| Female | 0.901 (0.887) |
| Income | 1.187 (0.167) |
| Education: Associate's or some college | 0.247 (0.218) |
| Education: Bachelor's or graduate degree | 0.671 (0.730) |
| Religion: non-Christian | 0.339 (0.400) |
| Religion: non-religious | 2.384 (0.344) |
| Religion: atheist | 0.740 (0.749) |
| Nationality: non-USA | 0.762 (0.858) |
| Ethnicity: Asian or Pacific Islander | 0.440 (0.485) |
| Ethnicity: other non-White | 0.835 (0.863) |
| Baseline odds | 19.525* (0.026) |
| Number of observations | 1067 |
| Number of participants | 516 |
| Wald $\chi^2$ | 11 df, 15.90 (0.145) |

Two-sided tests: * $p < 0.05$, ** $p < 0.01$, *** $p < 0.001$

The table reports odds ratios and $p$ values (in brackets) from a random-intercept logistic regression model.

## 5. Between-individual and within-individual effects

We replicate the analyses in Table 1 with within-subject centering (van de Pol and Wright 2009) in order to separate between-individual effects from within-individual effects. Between-individual effects represent time-invariant differences in "types" of participants, e.g. seeds and invitees. Within-individual effects refer to changes over time in a "representative" individual as this participant receives or observes additional invitations. The between-individual values were calculated by averaging the manipulation over all of the observations for a particular individual. The within-individual values were calculated by taking the deviation of the manipulation in the focal observation from the individual's mean manipulation (i.e. the between-individual value). Thus, participants who interacted only once did not contribute to the calculation of within-individual effects.

The results reported in Table S5 reveal that the within-individual effects are generally stronger than the between-individual effects. Most strikingly, the effect of GR is entirely due to increased odds of donation among former seeds who become invitees and not due to time-invariant between-individual differences in behavior. In other words, participants needed to experience both the "seed" and "invitee" condition in order to activate conditionally generous behavior. Similarly, the effect of observation was more pronounced among participants who observed different levels of generosity in subsequent interactions. These results may be due to the fact that the experiment involved minimal GR and TPI stimuli, which might have become more prominent with repeated interaction.

**Table S5. Odds ratios for donating across treatments with disaggregated between-individual and within-individual effects.**

| Manipulation | A) GR | B) TPI+ | C) TPI– | D) GR x TPI |
|---|---|---|---|---|
| Invitee (receives a donated invitation) | | | | 0.948 (0.970) |
| Between individuals | 0.692 (0.814) | | | |
| Within individuals | 15.238** (0.008) | | | |
| Has previously received donated invitations | 1.017 (0.985) | | | 0.760 (0.782) |
| Seeds | | | | |
| Observes 0-75 | | | | |
| Between individuals | | 4.428 (0.295) | (baseline) | (baseline) |
| Within individuals | | 140.502* (0.035) | | |
| Observes 76-150 | | | | |
| Between individuals | | 1.974 (0.667) | 0.483 (0.809) | 0.655 (0.862) |
| Within individuals | | 7.170 (0.235) | 0.007 (0.197) | 0.041 (0.082) |
| Observes 151+ | | | | |
| Between individuals | | 0.280 (0.419) | 0.011 (0.290) | 0.059 (0.243) |
| Within individuals | | -- | 0.000 (0.207) | 0.003* (0.026) |
| Invitees | | | | |
| Observes 0-75 | | | | (baseline) |
| Observes 76-150 | | | | |
| Between individuals | | | | 23.012 (0.211) |
| Within individuals | | | | 74.650 (0.051) |
| Observes 151+ | | | | |
| Between individuals | | | | 5.125 (0.515) |
| Within individuals | | | | 1200.184* (0.018) |
| High payment | 77.251** (0.005) | 2.586 (0.287) | 0.881 (0.944) | 3.527 (0.290) |
| Time waited (in hours) | 0.972* (0.025) | 0.994 (0.645) | 1.032 (0.486) | 0.974 (0.081) |

| | | | | |
|---|---|---|---|---|
| Previous participations | 0.517<br>(0.147) | 0.876<br>(0.794) | 3.618<br>(0.550) | 0.415<br>(0.172) |
| Baseline odds | 16.470*<br>(0.049) | 4.805<br>(0.122) | 15.529<br>(0.377) | 121.539*<br>(0.015) |
| Number of observations | 516 | 371 | 175 | 554 |
| Number of participants | 252 | 277 | 133 | 266 |
| Wald $\chi^2$ | 6 df, 13.84*<br>(0.032) | 8 df, 6.87<br>(0.551) | 7 df, 2.30<br>(0.942) | 13 df, 11.42<br>(0.575) |

Two-sided tests: * $p < 0.05$, ** $p < 0.01$, *** $p < 0.001$

The table reports odds ratios and *p* values (in brackets) from random-intercept logistic regression models for A) seeds and invitees in the no-observation treatment by number of donated invitations received; B) seeds in the observation and no-observation treatments by number of donated invitations observed; C) seeds in the observation treatment by number of donated invitations observed; and D) seeds and invitees in the observation treatment by number of donated invitations observed by invitees compared to seeds.

## 6. Robustness by payment

To test the results for robustness by payment, we replicated the analyses in Table 1 separately for the low-payment condition (Table S6) and the high-payment condition (Table S7). Since we halve the sample size, the statistical power decreases and the tests are no longer significant. Nevertheless, the direction of the GR and TPI effects is consistent across the two payment conditions. The size of the effects varies but not significantly. Hence, we can conclude that there are no important differences in GR and TPI between the two payment conditions we investigated.

**Table S6. Odds ratios for donating across treatments for the low payment condition.**

| Manipulation | A) GR | B) TPI+ | C) TPI– | D) GR x TPI |
|---|---|---|---|---|
| Invitee (receives a donated invitation) | 4.493 (0.249) | | | 0.331 (0.400) |
| Has previously received donated invitations | 0.441 (0.511) | | | 0.899 (0.922) |
| Seeds | | | | |
| Observes 0-75 | | 23.889 (0.103) | (baseline) | (baseline) |
| Observes 76-150 | | 5.486 (0.324) | 0.038 (0.285) | 0.304 (0.444) |
| Observes 151+ | | 0.392 (0.621) | 0.000 (0.080) | 0.030 (0.093) |
| Invitees | | | | |
| Observes 0-75 | | | | (baseline) |
| Observes 76-150 | | | | 5.427 (0.353) |
| Observes 151+ | | | | 34.583 (0.129) |
| Time waited (in hours) | 0.945* (0.022) | 0.979 (0.296) | 1.062 (0.419) | 1.010 (0.620) |
| Previous participations | 0.740 (0.612) | 0.628 (0.437) | 0.864 (0.956) | 0.566 (0.410) |
| Baseline odds | 20.428* (0.047) | 7.707 (0.129) | 801.230* (0.046) | 45.870* (0.015) |
| Number of observations | 248 | 205 | 93 | 278 |
| Number of participants | 126 | 143 | 64 | 129 |
| Wald $\chi^2$ | 4 df, 5.99 (0.200) | 5 df, 4.16 (0.526) | 4 df, 4.51 (0.341) | 8 df, 7.80 (0.453) |

Two-sided tests: * $p < 0.05$, ** $p < 0.01$, *** $p < 0.001$

The table reports odds ratios and *p* values (in brackets) from random-intercept logistic regression models for A) seeds and invitees in the no-observation treatment by number of donated invitations received; B) seeds in the observation and no-observation treatments by number of donated invitations observed; C) seeds in the observation treatment by number of donated invitations observed; and D) seeds and invitees in the observation treatment by number of donated invitations observed by invitees compared to seeds.

**Table S7. Odds ratios for donating across treatments for the high payment condition.**

| Manipulation | A) GR | B) TPI+ | C) TPI– | D) GR x TPI |
|---|---|---|---|---|
| Invitee (receives a donated invitation) | 22.443* (0.049) | | | 0.323 (0.680) |
| Has previously received donated invitations | 1.351 (0.814) | | | 2.843 (0.766) |
| Seeds | | | | |
| Observes 0-75 | | 5.543 (0.263) | (baseline) | (baseline) |
| Observes 76-150 | | 0.357 (0.499) | 0.000 (0.172) | 0.000 (0.062) |
| Observes 151+ | | 0.108 (0.304) | 0.000 (0.308) | 0.000 (0.212) |
| Invitees | | | | |
| Observes 0-75 | | | | (baseline) |
| Observes 76-150 | | | | 92352.67 (0.054) |
| Observes 151+ | | | | 27786.6 (0.335) |
| Time waited (in hours) | 0.998 (0.922) | 1.010 (0.649) | 1.011 (0.852) | 0.837** (0.002) |
| Previous participations | 0.704 (0.610) | 1.491 (0.701) | 25.515 (0.458) | 0.095 (0.247) |
| Baseline odds | 40.645 (0.056) | 6.214 (0.247) | 46799.88* (0.016) | $1.75 \times 10^{12}$*** (0.000) |
| Number of observations | 268 | 166 | 82 | 276 |
| Number of participants | 126 | 134 | 69 | 137 |
| Wald $\chi^2$ | 4 df, 4.40 (0.355) | 5 df, 2.81 (0.730) | 4 df, 2.66 (0.617) | 8 df, 11.92 (0.155) |

Two-sided tests: * $p < 0.05$, ** $p < 0.01$, *** $p < 0.001$

The table reports odds ratios and *p* values (in brackets) from random-intercept logistic regression models for A) seeds and invitees in the no-observation treatment by number of donated invitations received; B) seeds in the observation and no-observation treatments by number of donated invitations observed; C) seeds in the observation treatment by number of donated invitations observed; and D) seeds and invitees in the observation treatment by number of donated invitations observed by invitees compared to seeds.

## References

van de Pol, Martijn, and Jonathan Wright. 2009. “A simple method for distinguishing within- versus between-subject effects using mixed models.” *Animal Behaviour* 77: 753–758.
